# Effectiveness of a Program to Improve Attention towards Affective-Sexual, Bodily and Gender Diversity in University Students

**DOI:** 10.3390/ejihpe11040088

**Published:** 2021-10-04

**Authors:** Francisco Manuel Morales-Rodríguez

**Affiliations:** Department of Educational and Developmental Psychology, Faculty of Psychology, Campus Universitario de Cartuja, University of Granada, 18071 Granada, Spain; fmmorales@ugr.es; Tel.: +34-680-976-924

**Keywords:** affective-sexual, bodily and gender diversity, innovation, program, university students

## Abstract

It is necessary for the university environment to contribute to the improvement of the attention paid to affective-sexual, bodily, and gender diversity. This research deals with how, by means of a teaching innovation program, competences for affective-sexual diversity were developed. Specifically, negative attitudes towards diversity, knowledge, and degree of empathy on these issues before and after the implementation of the program are compared. The degree of satisfaction, perceived usefulness, and fulfillment of the objectives proposed in the program were also evaluated. An ex post facto design was used. The participants in this study were 129 students belonging to Educational Sciences and Psychology, out of 2400 who benefited from the innovation program. The results showed an increase in competences related to the attention to diversity, with the improvement of attitudes and knowledge about affective-sexual diversity after the application of the program. It is concluded that this type of innovation program, with quality training, contributes to the improvement of coexistence and the prevention of gender violence in university classrooms, eliminating stereotypes and negative attitudes towards diversity.

## 1. Introduction

In current undergraduate degrees, the acquisition and/or development of interpersonal skills by students is necessary. Specifically important are those skills related to education that attend to affective-sexual, bodily, and gender diversity, aligning with the Second Plan of Equality of the UGR [1]. Within this scenario of the European Higher Education Area (EHEA) [2], it is relevant to develop an innovative type of teaching program that provides students with high-quality training in this area.

As recently highlighted [3], these issues are becoming increasingly relevant in socio-legal terms—the role of Spanish universities with respect to affective-sexual, bodily, and gender diversity still needs to be analyzed. In fact, in Spain, there is a current debate regarding certain legislative provisions related to this issue. In that sense, [4] indicates precisely that the recession of COVID-19 (coronavirus disease) could increase discriminatory attitudes towards LGBTQIA+ (Lesbian, Gay, Bisexual, Transgender, Intersexual, Queer, Intersexual, Asexual) people and mental health problems due to the stress of minorities. In the same vein, [5] considers that the social isolation imposed by the COVID-19 pandemic brings to the fore, in an empowering way, some worrying indicators of domestic violence and family violence against women. Organizations addressing domestic violence have already seen an increase in domestic violence due to forced coexistence, economic stress, and fears about the coronavirus. To this end, it is necessary to develop training and innovation actions in favor of education for gender equality and affective-sexual, bodily, and gender diversity that contribute to the improvement of coexistence and the prevention of homophobic bullying. Another recent study [6] also highlights the marginalization and stigmatization that non-binary people can suffer, with the consequences that this entails.

There is still much to be done for gender equality and attention to affective-sexual diversity. Among the autonomous provisions that can be highlighted in Spain are Law 2/2014, of 8 July, comprehensively written for non-discrimination on the grounds of gender identity and recognition of the rights of transgender people in Andalusia [7]. More recently, Law 8/2017 argues for the rights of the LGBTI collective, as well as their families [8,9]. Additionally, the Order of 28 April 2015 [10] includes an action protocol on gender identity in the Andalusian educational system. It is of great importance to work on the prevention of gender stereotypes in educational institutions, the closest environment of the students. In such settings, this type of program is necessary, in which attitudes and perceptions by the student body towards gender diversity in different contexts are evaluated.

The concept of affective-sexual diversity is what allows individuals to distinguish the different sexual options that exist in society [11]. The concepts considered fundamental for understanding affective-sexual, bodily and gender diversity are those of sexual orientation, sex, gender role, and gender identity [12,13,14,15]. With respect to these terms, the following definitions are identified [12]. “Sexual orientation” refers to the desire and/or sexual attraction felt by people of the same sex or of other sexes. “Sex” is the set of biological characteristics that allow for male and female categorization of individuals by distinguishing between reproductive sex, chromosomal sex, hormones, and gonads. This implies that some human beings may share genetic and phenotypic characteristics of females and males that can be combined in many ways (such as in intersex individuals). “Gender identity” can be defined as an individual’s personal identification as male, female, transgender, or otherwise nonbinary. Nonbinary refers to an identification that is neither male nor female, and can include other more specific identities such as “agender,” “genderqueer,” “intermediate gender, gender neutral, or third sex.” The biological sex may match the gender identity (cisgender) or may not match (transgender or nonbinary), which leads to a wide variety of gender experiences. The so-called “gender role” is defined as society’s assigned beliefs and characteristics as inherently male or female. Gender roles have cultural connotations and implications as they serve as sets of norms, beliefs, values, attitudes, and ways of being and behaving in different situations proper to each sex according to society, further influenced by descriptive and prescriptive sex-based stereotypes.

There are different psychological theories to explain the learning process of sexual and gender identity, such as cognitive–social learning models and psychoanalytic theory. However, since it is not the objective of this work, we will briefly point out some of the specific theories that apply to affective-sexual and gender diversity.

The two primary theories underpinning the concepts under analysis and their possible implications are: (1) essentialism, which argues that sexual dimorphism creates fixed male and female identities which are innate in nature and provide unquestionable implications for people’s lives and expectations; and (2) queer theory, emphasizing the influence of the sociocultural relay with freedom for the expression of sexuality and gender [12]. Within this queer theory, we can point out the existence of pansexuality, which denies the binary characterization of sex and gender, and how it attempts to go beyond such labels. In this sense, as stated in the doctoral thesis of [16], the definition of sex from the queer theory perspective would be “a social, political and linguistic construction that should not be based on biological arguments” (p. 28).

One of the consolidated theories, for which there is even an evaluation scale, is the multidimensional theory of [17], in which sexual orientation is believed to evolve over time and a person can be situated anywhere between the extremes towards the other sex and the same sex. It also shows that an individual’s identified sexual orientation may be affected by sociocultural pressure in that person’s environment.

A recent study [18] shows the existence of attitudes of respect, tolerance, and acceptance towards the LGBT community (lesbian, gay, bisexual, transgender) in university students. However, as another study [19,20] indicates, further education on the topic of acceptance and respect is still necessary in order to allow for more flexible sexual diversity, as well as to contribute to improved self-acceptance and reduced stigma. Specifically, the latter study [20] makes a distinction between the following terms in regards to self-identified sexual orientation: (a) heterosexual (sexual attraction to people of the opposite sex); (b) homosexual (sexual attraction to people of the same sex); (c) bisexual (sexual attraction to men and women and people who are non-binary or do not identify as women or men); (d) asexual (no sexual attraction to any person); (e) pansexual (sexual attraction to people of any sex or gender); (f) demisexual (sexual attraction to people with whom they have a very strong emotional bond); (g) heteroflexible (sexual attraction to people of the opposite sex and sometimes attraction to people of the same sex); and (h) sapiosexual (sexual attraction to people with high intelligence). These authors found that, in a sample of young Spaniards aged between 18 and 25 years old, 26.1% identified with a non-heterosexual sexual orientation according to the terms described above; specifically, 12.8% identified themselves as bisexual, and women expressed greater sexual attraction to both sexes, while men identified more with homosexuality.

From a reflexive model using a qualitative methodology, such as interviews with open questions, life histories, and ethnography, other studies can be found which consider the importance of inclusion and respect for diversity, especially in the face of new challenges and changes in gender distinctions, and the tendency towards a less fixed sexual identity in both the social and clinical spheres [21,22,23,24,25]. As [26] shows, the rigidity of roles for men and women has consequences whether those roles are followed or not in terms of the formation of a positive “I,” a sense of belonging and in the links that are created or fail to be created with other people.

With regard to the perception of future teachers towards the LGBTQIA+ community, some studies [27] point to the awareness and willingness of trainee teachers in primary education and early childhood education to contribute to training and promoting acceptance of affective-sexual, bodily, and gender diversity in the classroom. Another work [28] studied the state of academic awareness surrounding intersexuality in early childhood, primary and secondary education, finding a lack of research and academic ignorance that demonstrates the difficulty in implementing programs and measures for improved diversity and inclusion. Other recent research [29] emphasizes the role of socio-educational factors in the promotion of inclusion in the university environment, as these factors have a significant impact on academic performance in the most marginalized populations.

With respect to the contributions by previous research, it should be noted that there are more works that call for coexistence with regard to gender equality in secondary education, and even at this level, there are very few which focus on LGBTQ+ communities. However, there are still very few programs, such as the present one, aimed not only at education for gender equality, but also at education for affective-sexual, bodily, and gender diversity in the university environment, in which elements of innovation are introduced and activities are carried out transversally. Non-sexist language guides have been analyzed in this study, and students have also had the opportunity to participate in many joint activities such as workshops, seminars, congresses, conferences, scriptorium conferences, video forums, and exhibitions.

Taking the above into account, a successful teaching innovation program was coordinated and developed within the university curriculum (Plan FIDO UGR, Plan de Formación e Innovación Docente de la Universidad de Granada, PIE419, 2018–2020) [30], which aimed to raise awareness and promote education for sexual, bodily, and gender diversity (sexual orientations, gender expression and identity, and diverse characteristics), non-sexism, co-education, respect for gender identity, and the importance of prevention and its awareness in the university environment.

A multidisciplinary group of 25 teachers from various academic disciplines participated. Their students benefited from transversal and specific activities in favor of education for sexual, bodily, and gender diversity. These activities were adapted and blended into the contents of respective teaching programs. The implementation and adaptation of advanced learning methodologies encouraged autonomous learning, active participation, and the use of information and communication technologies (ICT) to raise awareness of these issues in university classrooms.

The project was developed over two academic years. The first year was aimed at raising awareness and training students with crosscutting and subject-specific activities. In the second year, together with the previous activities, joint activities were developed with the voluntary participation of students trained in the previous academic year.

The following transversal activities were developed within every subject area:

- Using questionnaires focusing attention on affective-sexual, bodily, and gender diversity;
- Developing language and guides (language and content, including references, resources, and materials) for each subject;
- Making LGBT people (and those working for LGBT rights) visible;
- Debating the importance of initiatives designed to promote respect towards affective-sexual, bodily, and gender diversity in the world today in Spanish and Andalusian schools, with subsequent reflective activities and the possibility of extending these debates to schools at the level of compulsory secondary education (Enseñanza Secundaria Obligatoria; ESO);
- Getting the participating students to design everyday life activities for the promotion of affective-sexual, bodily, and gender diversity.

The following specific activities were also carried out: Completing a specific activity based on the corresponding material or subject matter. The teachers participating in each educational activity, which was linked to affective-sexual, bodily, and gender diversity, orientation, and professional ethics, selected the specific activities that would be exclusively carried out within each subject area. These were based on the pedagogical criteria and suitability of the methodology to the content and objectives of the activity. Examples of possible activities included: Watching a short or long-format film followed by a discussion and a debate; a lecture by an expert in the field and completion of follow-up questions on the virtual campus (designed for this purpose); participation in national and international debate forums; construction of a Wiki, chat, or forum for discussion and its use to solve a particular problem or obtain suggestions for everyday ideas to encourage the equality of gender differences; a video or book forum; creation of a glossary of terms related to these issues; asking students to find an LGBT-supportive news item followed by critical reflection upon it in class; creation of a bibliographic file; and analysis of a text.

The student body also benefited from the following joint activities: (a) Commemoration of anniversaries (performance, concert, slogans/stickers for safe spaces); attendance and participation in the *1st Conference on Attention to Affective-Sexual Diversity* held at the Faculty of Psychology and the *1st International Congress on Attention to Affective-Sexual Diversity* held at the Faculty of Psychology; and the *1st International Congress on Attention to Affective-Sexual Diversity* held in Andalusia; (b) Interdisciplinary round tables, such as for the analysis of laws and their development, depathologization, and families; research in education and health. Participation of two experts and other autonomous communities; Legislation and public policies. Participation of two experts and the Andalusian community; Experiences in ESO (Compulsory Secondary Education, Vocational Training, Infant and Primary Education, Language Schools).

The objectives of the present study were: (a) To analyze the descriptive statistics as well as Student’s T-test comparing the initial data at the beginning of the program with the final data once the innovation program had been completed with a series of variables selected to control the effect of the program; (b) To evaluate the degree of satisfaction and perceived usefulness of the activities of the innovation program; (c) To examine the degree of perceived usefulness of the activities carried out to fulfill the objectives of the project; (d) To examine the relationships between the variable attitudes towards equality and affective-sexual diversity and other psychoeducational variables, such as styles of thinking; and e) To assess the degree of fulfillment of the objectives of the teaching innovation program by the teaching staff and students.

### Research Hypothesis

An increase in the development of socio-emotional skills, such as empathy, is expected to decrease negative attitudes towards affective-sexual, bodily, and gender diversity after the development of the activities of the innovation program. It is also expected that the knowledge about affective-sexual diversity will be greater after the training received in the program.

## 2. Materials and Methods

### 2.1. Participants

The innovation project benefited 2400 university students from different faculties (such as Educational Sciences, Psychology, Social Work, Law, and Philosophy and Letters) who carried out the proposed activities. A total incidental sample of 129 students belonging to the Faculty of Educational Sciences and the Faculty of Psychology of the University of Granada were selected. Most of them belonged to the first year of the Degree in Primary Education and the Degree in Psychology. Of the 129 students, 69% were women. In terms of marital status, most of them were single. They were aged between 18 and 25 years, with an average age of 20.87 years old. A control group was also used with a sample of 95 students (67% female) from groups that did not participate in the program but belonged to the same faculties. A multidisciplinary group of 25 teachers from different areas of knowledge participated. The present study protocol was approved by the Ethics Committee of the University of Granada (Granada, Spain), with the registration number: 2056/CEIH/2021. The participants completed an individual informed consent form to participate in the study.

### 2.2. Instruments

#### 2.2.1. Instruments Selected to Monitor the Effect of the Program

Adaptation of School Doing Gender/Students—Scale of Student Attitudes towards Co-education [31]: This instrument evaluates student attitudes towards co-education and the construction of a new gender culture in schools based on equality between people, according to a 5-point Likert-type scale with 1 = Completely disagree and 5 = Completely agree. It presents adequate psychometric properties of reliability and validity. Cronbach’s alpha (α) for the current study for the scale was 0.92.

Negative Attitudes towards Trans People Scale (NATS) [32]. This instrument consists of nine items. The total score was obtained by adding scores according to a 5-point Likert scale ranging from 1 = Strongly disagree to 5 = Strongly agree. This scale has an excellent internal consistency in the study sample (α = 0.88). Higher scores are indicative of a higher level of negative attitudes towards trans people. Some items in this instrument include: “Trans people should not be allowed to teach in schools”; “trans people tend to be sexually promiscuous”; and “trans people are more likely than the rest of society to get a sexual disease.” This instrument has previously been used in the Spanish population [33].

Modern Homophobia Scale [34]. This instrument consists of 22 items in the subscale Homophobia toward gay men (MHS-G) and 24 items in the subscale Homophobia toward lesbians (MHS-L) with five-step response format: 1 = Strongly disagree, 2 = Somewhat disagree, 3 = Neither agree nor disagree, 4 = Somewhat agree and 5 = Strongly agree. A higher score indicates a more positive attitude to homosexuality, which is evidenced by a more positive attitude toward lesbians and gays. The internal consistency for the scale for the current study was α = 0.87. This test has been validated in the Spanish language for use in Spanish populations [35].

Cognitive and Affective Empathy Test (TECA) [36]. This test instrument comprises 33 items that are rated on a Likert scale from one (strongly disagree) to five (strongly agree). It assesses cognitive-affective skills related to the level of empathy regarding diversity-related issues. The test consists of four dimensions. Within the cognitive area are the dimensions of perspective-taking and emotional understanding. The affective area includes empathic stress and empathic joy. It presents adequate psychometric properties of reliability and validity in the study sample (α = 0.88). This test has been used in numerous studies involving the Spanish population (e.g., [37]).

Stenberg–Wagner Thinking Styles Questionnaire [38,39] (Sternberg, 1994, short form). This questionnaire contains 65 items with a seven-step response format: 1 = Not at all (if the statement does not fit at all); 2 = Almost, 3 = Slightly; 4 = A little; 5 = Quite a lot; 6 = A lot; 7 = Completely. It evaluates individual preferences for task performance, project development, and mental processes. It is made up of 13 scales of five items each: Legislative, executive, judicial, local, global, liberal, conservative, hierarchical, monarchical, oligarchical, anarchical, internal, and external. The internal consistency for the present sample ranges between 0.70 and 0.86. This questionnaire has previously been used within Spanish university populations [40].

#### 2.2.2. Instruments of Continuous Evaluation during the Development of the Program

Rubric answered by the students [30]. This rubric evaluated the degree of satisfaction and usefulness reported by the educational activities in favor of affective-sexual, bodily and gender diversity carried out by participants, according to a five-step scale with 1 = not at all and 5 = very much. It also contains a series of items related to knowledge about concepts and terminology related to affective-sexual diversity, knowledge about tools for the detection and prevention of LGBTI violence and harassment, beliefs about the need to receive training in affective-sexual, bodily, and gender diversity (ASCGD), detection of stereotypes, self-perceived socio-emotional competence to deal with these issues in the classroom as future teachers, and to face situations of discrimination and harassment in everyday life.

The rubric was answered by teachers and students for the evaluation of the degree of compliance with the objectives of the Program [30]. The evaluation was 1 = This objective has not been fulfilled at all; and 5 = Yes, this objective has been totally fulfilled.

The program and its methodology: Although described in the introduction, the general objectives of the advanced teaching innovation program titled “Transversal education for affective-sexual, bodily and gender diversity” (Code 419, Call for Teaching Innovation Projects and Good Practices of the FIDO UGR Plan 2018–2021) [30] were to raise awareness and promote education for sexual, bodily and gender diversity (sexual orientations, gender expression and identity and diverse characteristics), non-sexism, co-education, respecting gender identity, and the importance of prevention and its awareness in the university environment.

### 2.3. Design and Procedure

An ex post facto design was used. A cross-sectional non-experimental research design was implemented, and convenience sampling was used for selecting student participants. Participants were asked to complete a series of self-report Likert-response scales that were previously outlined. The Ethics Committee of the University of Granada approved the program and the studies derived from it. Evaluations were carried out before starting the program and after ending the program with different variables selected to record the effect of the program. Informed consent was requested, and the confidentiality of the data and the voluntary nature of participation were ensured with participants. The questionnaires were administered collectively with different groups of participants in different classrooms.

### 2.4. Data Analysis

For the data analysis, the computerized statistical package SPSS V. 22 was used. An examination (descriptive statistics and Kolmogorov–Smirnov normality tests) of the study variables was carried out to verify compliance with the assumptions of normality, non-collinearity, and Pearson’s correlation.

Descriptive analyses were carried out reflecting percentages, means, and standard deviations of the participating sample as well as Student’s T-test for related samples, to determine if there were statistically significant differences in the evaluation of the variables of the questionnaires, including negative attitudes towards transsexual people, empathy in the cognitive and affective dimension, and knowledge about equality and affective-sexual, bodily, and gender diversity.

To assess the degree of satisfaction and perceived usefulness associated with the program activities (completion of questionnaires and subsequent reflection on the results in class seminars; debate on the importance of education for affective-sexual diversity; drawing up a list of daily actions to be carried out in favor of affective-sexual, bodily and gender diversity; analysis of news on diversity; analysis and assessment of guides on non-sexist language; viewing of a film or short film about this subject; resources which attend to affective-sexual, bodily and gender diversity; carrying out a list of daily actions in favor of affective-sexual and gender diversity; analysis of news on diversity; analysis and evaluation of guides on non-sexist language; Carrying out a group activity (mural, etc.) related to diversity; and a conference or talk. The mean and standard deviation were extracted. Likewise, to evaluate the degree of fulfillment of the objectives of the innovation program it is also extracted the mean, the standard deviation, and extensive information available in print and on the Internet of qualitative comments that have not been presented in this work. Much more information that is not presented in this study is available in the platform used with the participants in the reports delivered, participations and comments in forums, wikis, and glossaries of terms, in addition to the interviews carried out.

Analyses of the relationships between the variables—attitudes towards gender equality and affective-sexual diversity and the variable thinking styles/cognitive flexibility—were carried out using Pearson’s correlation analysis.

## 3. Results

### 3.1. Differences in Empathy, Attitudes, and Knowledge about Affective-Sexual, Bodily, and Gender Diversity before and after Participation in the Innovation Program

Table 1 presents the analysis of the effects of the innovation program comparing the scores on the variable’s empathy, attitudes, and knowledge about affective-sexual, bodily, and gender diversity in this sample before and after their participation in the program. Differences in the scores on these variables were found. Specifically, an increase was found in the scores of the empathy variables in their cognitive and affective dimensions, as well as in the knowledge selected for their evaluation. A decrease in negative attitudes and stereotypes towards transgender people was also found.

Table 2 below shows an extract of the final data of the participants in the program referring to the degree of satisfaction and perceived usefulness obtained in the educational activities used in the program. Considering that the evaluation was from one to being, the average scores showed that the activities reported a high degree of satisfaction. Participants were also asked about the degree to which these activities contributed to the fulfillment of the objectives of the project, whose score was 4.90 for activity 7 (student body), 4.85 for activity 4 (student body), and 4.83 for activity 7 (Faculty), for example.

### 3.2. Relationships between the Variable Attitudes towards Affective-Sexual Diversity and Styles of Thinking

Table 3 shows the correlations between the variables of positive attitudes towards gender equality at the three levels (sociocultural, relational, and personal) and the different scales that are part of the thinking styles. The results show the existence of positive correlations between attitudes towards gender equality and the total score for the thinking styles variable.

### 3.3. Perceptions on the Degree of Compliance with the Objectives of the Project by the Teaching Staff, Students, and Association

Next, Table 4 presents the data referring to the degree of fulfillment of the project’s objectives by the teaching staff and students, and the perceived usefulness—the average values according to a five-step scale also ranging between 4 and 5.

## 4. Discussion

The general objective of this study was to evaluate the knowledge, attitudes in favor of diversity and gender equality, homophobia, transphobia, and the development of socioemotional skills, such as empathy before and after the implementation of the innovation program. The data supported the effectiveness of the teaching innovation program. The data were congruent with other previous programs on education regarding solidarity values and the development of empathy and prosocial behaviors that proved to be successful in university settings [41,42,43]. However, there are fewer programs focused on the development of competencies attending to affective-sexual diversity, and other aspects explained previously in university settings.

Regarding the objective related to the analysis of the relationship between attitudes towards gender equality with other psychoeducational variables, such as thinking styles, further research is needed. A previous study [44] demonstrated the existence of correlations between the variables’ attitudes towards gender equality and those of resilience and burnout. Comparably, a second study [45] indicated that women tend to be more empathetic than men, and that less empathetic people are more likely to experience behavior that is more violent, such as bullying in the school environment.

When considering the degree of satisfaction and usefulness of the activities, the participants reported a high degree of satisfaction, perceiving them as very useful. They helped them to acquire and develop knowledge, attitudes, and socio-emotional competences in this subject. For example, they developed empathy in the cognitive and emotional/socio-affective dimension to face certain situations. In this sense, participants perceived a high degree of compliance with the objectives of the innovation program. Likewise, they considered that the different types of educational activities carried out contributed in a highly effective and efficient way.

It is considered that the university environment constitutes one of the main agents of dynamization for education in gender equality and attention to diversity. Although continuous progress has been made in this area in terms of legislative provisions, such as the Valencian Strategy for Equal Treatment, Non-discrimination, and the Prevention of Hate Crimes 2019–2024, published on 6 February 2019, there is still an immense task to be completed regarding sexual diversity [46]. As [12] (2020, p. 17) states:

“There is an urgent need for sex education programs in schools and also at the community level (well-targeted media campaigns) that continue to normalize sexual diversity, but these programs should also be launched at a very early age, long before LGBTphobia has settled in people’s minds. Current programs are short, late, and with excessively limited content. We must go beyond the prevention of unwanted pregnancies or the prevention of STIs and start talking about healthy sexuality, affection, autoeroticism, enjoyment, sexual orientation, gender, and sexual freedom. It is essential that we pay more attention to the internalized forms of homo/bi/trans-phobia that can do so much damage to the mental health of the individual who deviates from the norm. We must make a greater effort to make female homosexuality visible, even among LGBTI groups. It is also urgent to take measures that help to reinterpret bisexuality both among the general population and among LGBTI groups themselves so that it is considered as another orientation and not as a state of indecision, transition, or cowardice.”

Along these lines, it is necessary that educational centers promote education for the improvement of coexistence and specifically in terms of training or education for affective-sexual diversity [12,47,48].

### Limitations of the Study and Future Line of Research

One limitation includes the need to employ a longitudinal design that would allow a more comprehensive follow-up than that carried out continuously during the two-year duration of the advanced innovation program. In the future, more robust multivariate designs could be utilized, where more variables or constructs are included, and, in particular, questionnaires with even more inclusive items could be used regarding the assessment of affective-sexual, bodily, and gender diversity.

Another limitation is the use of self-report measures. However, it can be noted that much more information that is not presented in this study is available in the platform used with the participants in the reports delivered, participations and comments in forums, wikis, and glossaries of terms, in addition to the interviews carried out.

A limitation of the article that must be declared is that the sample consisted of only students pursuing either a degree in primary education or a degree in psychology, since the effects of the program have been analyzed more readily in these two degrees, in which the design of the training plan could be integrated more easily across the different subjects. In other degrees, the training activities were carried out, but due to the enormous changes and adaptations generated by the COVID-19 pandemic, it was not possible to administer the questionnaires on as many occasions as in the aforementioned degrees. In this sense, the idea is to provide continuity for this program in the rest of the degrees, either with this same program or with adapted training activities. Likewise, data from other instruments that have been applied will be analyzed in order to continue evaluating the program’s impact.

Future research is needed from an inclusive perspective to fully capture the diversity of the LGBTQIA+ collective (lesbian, gay, bisexual, transgender, queer, intersex, asexual+). A more precise evaluation of sexual diversity is required of future teachers as well as students, with the construction and validation of new instruments for the specific evaluation of knowledge, procedures, and attitudes towards affective-sexual diversity.

This diversity training must be included in new subjects, even if they are optional, and existing curricula and subjects in social sciences, humanities, and health degrees must be able to introduce this type of content more explicitly. Sometimes it is only a question of rethinking or reinventing the same educational activities by introducing elements in favor of affective-sexual, bodily, and gender diversity education.

At a socio-community level, there is a need for more awareness and educational programs on affective-sexual diversity in order to aid the prevention of stereotypes and provide greater social support for people who, in this case, sometimes do not have their own support network (such as family) and may even experience internalized homophobia. Even more evidence is needed on the experiences of the wide range of LGBTQ-identified individuals and how their experiences affect them in order to offer necessary support and attention. For example, there are hardly any programs or studies on affective-sexual, bodily, and gender diversity focused on older people, or neurodivergent individuals.

In order to provide continuity to this work, in which digital technologies have played a fundamental role in the development of many of the program’s activities, an application (app) is being designed for affective-sexual education.

## 5. Conclusions

A positive effect of the program was drawn concerning the existence of statistically significant differences before and after the application of the program in the development of socio-emotional competence and for the attention to diversity. Specifically, attitudes and knowledge towards gender equality improved, negative attitudes towards transgender individuals decreased, and there was a clear development of cognitive and affective empathy. Correlations between attitudes towards gender equality and the total score for the thinking styles variable were positive. The program also shows that the participants were extremely satisfied and perceived their participation in this innovative program to be very useful. The teaching staff and participants perceived a very high degree of compliance with the objectives of the project as endorsed in the various reports submitted to the Innovation Unit at the university throughout the process. Statistically, significant correlations were also found between attitudes towards gender equality and the thinking styles variable. At present, it is relevant to continue with this type of innovation program aimed at the construction of coexistence, not only of knowledge in full debate on certain legislative provisions on affective-sexual, bodily, and gender diversity. It is necessary to avoid situations of harassment that still continue to aggrieve certain groups and that can be more serious due to the social and educational impact produced by the pandemic and the confinement necessitated by the coronavirus disease. Diversity education programs and the provision of coping strategies focused on emotional management and social support must be urgently implemented at the university level. Doing so will contribute to the prevention of violence towards the LGBTI collective, especially with regard to the stereotypes and stigma targeting bisexual, intersex, and transgender individuals which can lead to lower self-esteem and self-worth. These impact not only university students’ health, but also the teaching-learning process and student performance. The changes generated by the pandemic and the increased use of Information and Communication Technologies in all areas require competent school leaders to design training programs and activities whose messages aid in the prevention of violence and both overt and subtle discrimination against LGBTQ+ communities. University teaching staff, in collaboration with other educational agents, can significantly contribute to inclusion, diversity and human rights education related to sexual and gender diversity in a way that is more in line with the demands of current society. Likewise, the need for affective-sexual diversity to become more and more visible in mass and interpersonal media, as well as in prevention campaigns carried out by institutions, is also clear.

Numerous implications for practice and theory can be derived from this study, such as the fact that the obtained data can contribute to the design of more effective psychoeducational actions promoting the necessary social and educational inclusion in the university environment. This also leads to improved psychosocial well-being, self-acceptance of diversity, prevention of stereotypes and limiting beliefs contrary to equal opportunities, and more generally, the improvement of the climate and quality of life in higher education.

This work has practical and professional implications related to its contribution to the 2030 Agenda for Sustainable Development, assuming social responsibility for improving attitudes toward affective-sexual diversity, and the internalization of socio-emotional competences acquired in this program with self-perceptions that translate into less prejudice, LGBTphobia, and fewer violent and discriminatory behaviors towards the LGBTQ collective.

With this type of work, we are joining forces to develop more empathetic institutions that contribute to the normalization of diversity and the prevention of LGBTphobia, and advocating for a society more committed to the values of diversity, non-violence, acceptance, solidarity, respect for diversity and human rights. Educational centers must carry out more educational programs for the improvement of coexistence and prevention of violence. Some groups face alarming rates of violence, as in the case of transgender individuals, making the implementation of such programs for awareness and the development of necessary psychoeducational interventions that much more urgent.

In addition to generating knowledge, this type of program can contribute to normalizing affective-sexual diversity and improve social support, thereby reducing stigmatization that LGBTQ+ people, in particular, may suffer. Another major opportunity and resource derived from this study is the large amount of educational material that has been provided to future teachers, which will be used to promote diversity and foster more inclusive classrooms.

## Figures and Tables

**Table 1:** Comparison of pre-post means in the group of university students participating in the innovation program in the variables of the study.

	Student’s *t-Test*						
	*M*	*DT*	*M*	*DT*	*t*	*gl*	*p*
Negative attitudes towards transgender people	11.25	3.66	9.93	2.22	4.04	126	0.00
Adoption of perspectives	30.59	3.36	33.23	3.53	−26.67	126	0.00
Emotional understanding	31.46	5.29	34.15	5.29	−24.23	126	0.00
Empathic stress	23.39	3.85	25.53	3.82	−22.75	126	0.00
Empathetic joy	28.74	3.10	31.33	3.11	−29.48	126	0.00
Positive Beliefs	1.56	0.56	3.84	0.37	−22.14	126	0.00
Knowledge of equality and affective-sexual diversity	1.78	0.50	3.59	0.49	−24.35	120	0.00
Social-emotional competencies	1.34	0.64	3.90	0.30	−25.68	124	0.00

*gl*: degrees of freedom; *p*: significance level; *t*: Student’s *t-statistic* for dependent samples; *M*: mean; *SD*: standard deviation; *p*: critical level of significance.

**Table 2:** Degree of perceived usefulness and satisfaction with educational activities in favor of affective-sexual, bodily, and gender diversity. *Dt* = standard deviation.

Activities	Degree of Satisfaction Mean (dt)	Degree of Perceived Usefulness (Student) Mean (dt)	Degree of Contribution of These Activities to the Fulfillment of the Project Objectives (Student) Mean (dt)	Degree of Contribution of These Activities to the Fulfillment of the Project Objectives (Faculty) Mean (dt)	Degree of Perceived Usefulness (Faculty) Mean (dt)
1. Completion of questionnaires	4.00 (1.21)	4.36 (1.12)	4.49 (0.70)	4.50 (0.84)	5.00 (1.00)
2. Discussion on the importance of education for affective-sexual diversity	4.25 (0.87)	4.46 (0.80)	4.49 (0.70)	4.83 (0.41)	4.67 (0.82)
3. Elaboration of a list of daily actions to carry out in favor of affective-sexual bodily and gender diversity.	4.26 (0.83)	4.00 (0.77)	4.50 (0.70)	4.83 (0.41)	4.67 (0.52)
4. Diversity news analysis	4.33 (0.79)	4.39 (1.83)	4.85 (0.69)	4.67 (0.81)	4.83 (0.40)
5. Analysis and evaluation of guides on non-sexist language.	4.00 (0.83)	4.00 (0.83)	4.67 (0.79)	4.40 (0.89)	5.00 (0.00)
6. Viewing of a film or short film on this thematic line.	4.33 (0.75)	4.36 (0.83)	4.67 (0.65)	4.67 (0.81)	5.00 (0.00)
7. Resources provided on attention to affective-sexual, bodily and gender diversity.	4.00 (0.60)	4.66 (0.67)	4.90 (0.51)	4.83 (1.03)	4.33 (1.03)
8. Carrying out a group activity (mural, etc.) on attention to diversity.	4.08 (0.90)	4.48 (0.87)	4.68 (0.75)	4.33 (1.91)	4.67 (0.51)
9. Conference or lecture	4.00 (1.04)	4.5 (1.10)	4.80 (0.69)	4.35 (1.03)	4.55 (1.00)

**Table 3:** Correlation between attitudes towards gender equality and thinking styles in university students.

	1	2	3	4	5	6	7	8	9	10	11	12	13
Attitudes towards the equality of gender (total)	−0.29	−0.27	0.45 *	−0.06	0.17	0.11	0.11	−0.19	0.12	0.12	0.06	0.03	0.11
Sociocultural level	0.26	0.22	0.46 *	0.07	0.28	0.08	0.18	−0.18	−0.01	0.02	0.07	0.15	−0. 03
Relational level	0.41 *	0.46 *	0.33	0.14	0.08	0.15	0.04	−0.33	−0.14	0.29	0.14	0.04	−0.32
Personal level	−0.09	−0.26	−0.04	−0.14	0.12	−0.07	0.11	−0.03	0.11	−0.21	−0.14	0.02	−0.33

Note. 1. Legislative; 2. Executive; 3. Judicial; 4. Local; 5. Global; 6. Liberal; 7. Conservative; 8. Hierarchical; 9. Monarchical; 10. Oligarchical; 11. Anarchical; 12. Internal, and 13. External; * *p* < 0.05.

**Table 4:** Degree of compliance with the project objectives (1 = totally disagree; 7 = totally agree). Mean and standard deviation.

	Student Body (Mean and Standard Deviation)	Faculty (Mean and Standard Deviation)
Objectives:		
GENERAL:		
- Sensitize and raise awareness towards inclusive education about affective-sexual, bodily, and gender diversity in university environments.	4.3 (1.41)	4.66 (0.51)
- Promote among students theoretical-practical knowledge about inequality, discrimination, and the value of equality, gender equity, co-education, and respect for different gender, sexual, and bodily identities.	4.14 (1.06)	4.17 (0.41)
- Promote transversality for the development of inter- and intra-personal skills through the collaboration and joint participation of students and university teaching staff from different degree courses and teaching areas.	4.4 (1.05)	4.33 (1.21)
- Reflect upon the importance of intersectionality for the promotion and mobilization of rights in the area of equality and justice, linking the situations of discrimination that they produce with their specific socio-political, legal, and economic contexts.	4.40 (1.06)	4.00 (1.89)
SPECIFIC JOINT OBJECTIVES:		
- Encourage contact with associations and social organizations that work to favor the support of, and attention to, sexual-affective diversity and sexual orientation.	4.50 (1.06)	4.33 (0.51)
- Reflect, from different perspectives (education, health, social services, and justice), upon situations in which discrimination based on gender or sexual orientation can be observed in everyday life.	4.22 (1.21)	4.50 (0.55)
- Promote daily habits that favor affective-sexual, bodily, and gender diversity.	4.47 (1.02)	4.83 (0.41)
- Introduce practical activities to draw attention to sexual, bodily, and gender diversity as an everyday part of university life.	4.34 (1.07)	4.56 (0.98)
- Make visible and analyze the values derived from the Spanish Constitution and Spanish laws that allude to sexual, bodily, and gender diversity.	4. 29 (1.05)	4.80 (0.40)
- Motivate students to carry out activities to raise awareness of affective-sexual, bodily, and gender diversity.	4.14 (0.89)	4.67 (0.51)
- Reflect upon how ethics affect professional life, also emphasizing measures taken to prevent stereotypes and promote respect for sexual, bodily, and gender identities.	4.14 (0.69)	4.80 (0.45)
SPECIFIC OBJECTIVES BY AREA OF KNOWLEDGE:		
Education:		
Promote respect towards bodily, sexual, and gender identities in different educational stages.	4.79 (1.06)	4.20 (0.44)
Understand the fundamental aspects of sexual-affective, bodily, and gender diversity.	4.68 (1.41)	4.00 (1.23)
Reflect, from the perspective of different educational stages (university, primary, secondary, or adult education), upon situations involving discrimination due to gender, bodily identity, or sexual orientation in day-to-day life.	4.58 (1.24)	3.60 (1.63)
Analyze the impact of different types of music, genres, or musical styles in terms of education about gender and bodily identity, and the equality of the diverse affective-sexual orientations they refer to.	3.86 (1.06)	4.20 (0.83)
Promote knowledge and activities contributing towards the development of bodily, sexual, and gender identity in early childhood and primary and secondary education.	4.14 (0.89)	4.80 (0.44)
Recognize bullying and discrimination in education as a result of sexual, bodily, or gender diversity.	4.00 (1.00)	4.40 (0.54)
Dismantle myths about the educational style and development of children in non-heteronormative families.	3.85 (1.09)	3.80 (1.09)
Examine the regulations against school harassment, gender equality, and sexual, bodily, and gender diversity at educational centers.	4.14 (0.89)	4.00 (1.73)
Analyze the relationships between child mistreatment and affective-sexual, bodily, and gender diversity.	4.14 (0.89)	3.60 (1.51)
Promote attention towards the factors associated with these relationships within child mistreatment intervention programs.	4.42 (0.53)	3.80 (1.65)
Be aware of the need to prevent behaviors of rejection or homophobia in the educational environment and among minors, and the desirability of empathizing with people’s right to diversity and emotionally supporting each individual’s choices.	4.00 (0.81)	3.70 (1.34)
Musical expression:		
Reflect upon the impact of both traditional and current songs in the configuration of attitudes and prejudices regarding gender equality and equality of affective-sexual diversity (the use of ICT will be essential here).	4.11 (0.89)	3.40 (1.51)
Approach to non-normative musical expressions: queer tango, queer songs, transgender music, transvestism in the opera, etc.	3.99 (1.02)	3.60 (0.89)
Trips to musical events related to the project’s themes.	3.85 (1.06)	3.60 (0.89)
Justice/Rights:		
Analyze different legislative provisions that form part of the transversal regulations, as well as international, educational, national, international, and regional rules.	3.71 (1.12)	3.50 (1.29)
Promote respect for bodily, sexual, and gender identities among those with a direct relationship with the fields of the Law (the judiciary, legal professionals, etc.).	4.00 (1.41)	4.25 (0.50)
Reflect upon everyday life situations that embody the importance of physical, sexual, and gender diversity, as well as discrimination.	4.28 (1.11)	4.00 (1.27)
Think about the possibility of a legal system that recognizes bodily, sexual, and gender identity.	3.85 (1.06)	3.50 (1.00)
Carry out a comparative study of the different regulations currently in place globally in relation to bodily, sexual, and gender diversity.	3.87 (1.23)	3.50 (1.29)
Health:		
Identify the dynamics of pathologization and human rights violations in health fields.	3.85 (0.89)	3.75 (1.26)
Encourage the promotion of respect for sexual, bodily, and gender diversity in healthcare.	3.71 (0.95)	3.00 (1.81)
Development of strategies for clinical practices based on depathologization and human rights.	3.22 (0.69)	4.33 (0.81)

## Data Availability

The data could be requested by the scientific community in the ethical terms to be determined.
